# Mechanism and Effect of Temperature on Variations in Antibiotic Resistance Genes during Anaerobic Digestion of Dairy Manure

**DOI:** 10.1038/srep30237

**Published:** 2016-07-22

**Authors:** Wei Sun, Xun Qian, Jie Gu, Xiao-Juan Wang, Man-Li Duan

**Affiliations:** 1 College of Natural Resources and Environment, Northwest A&F University, Yangling, Shaanxi 712100, China

## Abstract

Animal manure comprises an important reservoir for antibiotic resistance genes (ARGs), but the variation in ARGs during anaerobic digestion at various temperatures and its underlying mechanism remain unclear. Thus, we performed anaerobic digestion using dairy manure at three temperature levels (moderate: 20 °C, mesophilic: 35 °C, and thermophilic: 55 °C), to analyze the dynamics of ARGs and bacterial communities by quantitative PCR and 16S rRNA gene sequencing. We found that 8/10 detected ARGs declined and 5/10 decreased more than 1.0 log during thermophilic digestion, whereas only four and five ARGs decreased during moderate and mesophilic digestion, respectively. The changes in ARGs and bacterial communities were similar under the moderate and mesophilic treatments, but distinct from those in the thermophilic system. Potential pathogens such as Bacteroidetes, Proteobacteria, and *Corynebacterium* were removed by thermophilic digestion but not by moderate and mesophilic digestion. The bacterial community succession was the dominant mechanism that influenced the variation in ARGs and integrons during anaerobic digestion. Thermophilic digestion decreased the amount of mesophilic bacteria (Bacteroidetes and Proteobacteria) carrying ARGs. Anaerobic digestion generally decreased the abundance of integrons by eliminating the aerobic hosts of integrons (Actinomycetales and Bacilli). Thermophilic anaerobic digestion is recommended for the treatment and reuse of animal manure.

As an emerging contaminant, antibiotic resistance genes (ARGs) have become a great public health concern^1^,^2^,^3^. In particular, animal manure is an important reservoir of antibiotics and ARGs due to the overuse of antibiotics in the livestock industry^4^,^5^,^6^. Accumulating evidence suggests that the increased antibiotic resistance in bacterial pathogens is related to the application of manure and manure-derived fertilizer to land^7^,^8^,^9^. Thus, biotechnologies such as anaerobic digestion and aerobic compost may be employed as remedial measures to reduce the abundance of ARGs and pathogens prior to the application of animal manure to land^10^,^11^,^12^.

A few studies suggest that anaerobic digestion can potentially eliminate some of ARGs^13^,^14^,^15^. The mesophilic anaerobic digestion could significantly reduce cefazolin resistant bacteria^14^, and ARGs ermB, aphA2 and bla_TEM-1_^15^. The relative abundances (RAs) of *tet* and *erm* in swine manure declined by 1–3 orders of magnitude during anaerobic storage^12^. Temperature is an important parameter during anaerobic digestion and changes in temperature can have variable effects on biogas production^16^, ARGs^17^, and microbial community succession^18^. Diverse results have been obtained in various studies of the effects of temperature on ARGs during anaerobic digestion. Thus, Ghosh *et al*.^13^ and Diehl and LaPara^17^ suggested that thermophilic digestion performed better at reducing ARGs than mesophilic anaerobic digestion. Ma *et al*.^19^ found that thermophilic digestion was more effective than mesophilic digestion at decreasing the *ermB*, *ermF*, *tetO*, and *tetW* genes, but its performance was similar or worse in decreasing other ARGs and *intI1*. A recent metagenomic analysis by Zhang *et al*.^20^ detected no obvious differences in the total ARGs under thermophilic and mesophilic digestion. It should be noted that all of these studies were conducted using wastewater sludge, the effects of temperature on ARGs in animal manure were not studied. Due to the huge differences in physicochemical properties, antibiotic types and concentrations, and microbial community composition in wastewater sludge and animal manure^21^,^22^,^23^, it is highly likely that different effects on ARGs may be obtained by the anaerobic digestion of livestock manure. In addtion, the dynamics of ARGs during anaerobic digestion have received little study, and the underlying mechanism responsible for the variation in ARGs are not fully revealed.

Bacteria are the main carriers of ARGs and the changes in the bacterial community may lead to variations in the abundance of ARGs. Thus, we hypothesized that changes in the bacterial community comprise the main mechanism responsible for variation in ARGs during anaerobic digestion, where different temperatures will shape distinct bacterial communities with diverse effects on ARGs. The objectives of the present study were: (1) to compare the variation of ARGs in dairy manure under moderate (20 °C), mesophilic (35 °C), and thermophilic (55 °C) anaerobic digestion; and (2) to explore the relationships among the chemical properties, bacterial communities, and ARGs during anaerobic digestion. The results of this study may help to enhance the reduction of ARGs from animal manure by anaerobic digestion, thereby reducing the environmental risk due to ARGs.

## Material and Methods

### Experimental setup

The dairy manure used in this study was sampled from a medium-sized farm in Yangling, China. The digestion reactor (Figure S1) comprised 39 identical 250 mL triangular flasks each with an assistant bottle to maintain anaerobic environment. The working volume of each reactor is 200 mL. The digestion system contained 16 g of dairy manure and 40 mL of inoculum. The inoculum was obtained by a pre-anaerobic digestion for 30 days at 35 °C using the dairy manure. Each of the digestion reactors was placed in a constant temperature shaker, which was set at 160 rpm, with temperatures of 20 °C (moderate), 35 °C (mesophilic), or 55 °C (thermophilic). Each treatment was repeated in triplicate. The characteristics of the dairy manure and inoculum are shown in Table S1.

### Sample collection

The three prepared digestion mixtures were sampled immediately as the day 0 samples. Three flasks for each treatment were then sampled as triplicates on days 3, 12, 30, and 60. The digestion mixture samples were transferred to centrifuge tubes and centrifuged for 15 min at 5000 rpm. The supernatant was used to analyze the pH, available nitrogen (AN) content, soluble chemical oxygen demand (SCOD), and volatile fatty acid (VFA) contents. The precipitate was freeze-dried using a vacuum freeze dryer (Songyuan, China), ground to 1 mm with an ultra-centrifugal mill (Retsch Z200, Germany), and stored at −80 °C for DNA extraction.

### Determination of chemical properties

The pH was determined using a pH meter (Mettler Toledo, Switzerland). The AN and SCOD were determined with a flow injection analyzer (Westco Scientific, USA) and AQ4001 COD analyzer (Thermo Orion, USA), respectively. The concentrations of VFAs, including acetate, propionate, isobutyrate, butyrate, isovalerate, and valerate, were obtained by gas chromatography (Shimazu GC2010, Japan)^24^.

### DNA extraction and quantitative PCR (qPCR)

DNA was extracted from 100 mg of each sample using a FastDNA SPIN Kit for Soil (MP Biomedicals, USA), according to the manufacturer’s instructions. During DNA extraction, 5.5 M guanidinium isothiocyanate (Amesco) was added to remove humic acid.

Eleven tetracycline resistance genes (*tet*: *tetA, tetB, tetC, tetE, tetG, tetM, tetO, tetQ, tetT, tetW*, and *tetX*), five sulfonamide resistance genes (*sul*: *sul1, sul2, sulA, dfrA1*, and *dfrA7*), four fluoroquinolone resistance genes (*gyrA*, *par*C, *qnr*C, and *qnr*S), and two integrase genes (*intI1* and *intI2*) were analyzed by PCR and agarose electrophoresis. The detected ARGs and 16S rRNA gene were analyzed further by qPCR. The qPCR reaction mixture comprised 1 μL of DNA template, 0.25 μL of each 20 pM primer (ShengGong, China), 10 μL of SuperReal PreMix Plus (TianGen, China), and 8.5 μL of RNase-free water. The qPCR conditions comprised an initial hold for 15 min at 95 °C, followed by 40 cycles for 10 s at 95 °C, 20 s at the annealing temperatures shown in Table S2, and then 32 s at 72 °C. To eliminate the effects of inhibitory compounds, the DNA template was a tenfold dilution of extracted DNA. The qPCR was performed using Bio-Rad IQ5 (Bio-Rad, USA). The quantitative limit of qPCR was 10^4^ copies/g solid. Melting curve analysis was used to detect nonspecific amplification.

### 16S rRNA gene sequencing

The 16S rRNA gene high-throughput sequencing was performed by Realbio Genomics Institute (Shanghai, China) using the Illumina MiSeq platform. The 16S V3-V4 region was amplified using the primers U341F (ACTCCTACGGGAGGCAGCAG) and U806R (GGACTACHVGGGTWTCTAAT). The raw data were then subjected to a quality control procedure using UPARSE^25^. USEARCH was used to filter chimeras and the remaining sequences were clustered to generate operational taxonomic units (OTUs) at the 97% similarity level^26^. A representative sequence of each OTU was assigned to a taxonomic level in the RDP database using the RDP classifier^27^. To eliminate the differences caused by various sequencing depth among samples, the least number of obtained sequences (n = 33054) were randomly picked from each sample and used for subsequent bioinformatics analysis.

### Statistical analysis

Variance analyses were performed (ANOVA, least significant difference test, *P* < 0.05) and Pearson’s correlation coefficients were calculated using PASW Statistics 19.0. Principal components analysis and heatmap analysis were conducted with R3.1.0. Redundancy analysis was performed using CANOCO 4.5.

## Results

### AAs of ARGs after anaerobic digestion

**Figure 1:**
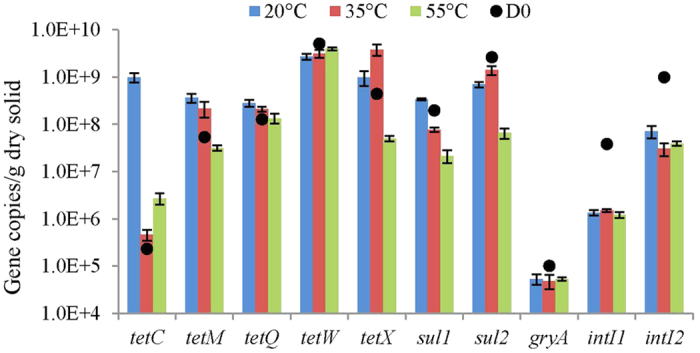
Absolute abundance of the ARGs and integrase genes detected at the start and end of anaerobic digestion. Black solid circles represent the absolute abundance of gene copies at the start of digestion. Values represent the means based on three replicates. Bars represent standard errors.

Ten ARGs and integrase genes were detected in dairy manure during anaerobic digestion (Fig. 1). The *tetW*, *sul2*, and *intI2* genes were most abundant with AA > 10^9^ copies/g dry solid in the initial digester. Excluding *tetC* and *gyrA*, the AAs of ARGs were >10^7^ copies/g dry solid in the initial digester. Four of five tetracycline resistance genes (*tetC*, *tetM*, *tetQ* and *tetX*) were enriched under the moderate and mesophilic treatments. By contrast, only *tetC* was enriched with the thermophilic treatment, *tetQ* had no significant change and the others declined by 0.1–1.5 log. The AAs of *sul1* and *sul2* declined under all treatments, except *sul1* was enriched with the moderate treatment, and the thermophilic treatment had the highest redution for these two genes (both > 1.0 log). The AA of *gyrA* declined by 47.3% after digestion and there were no significant differences among the three treatments. All of the treatments significantly decreased *intI1* and *intI2* by 1.2–1.5 log. Eight of 10 detected ARGs declined and five decreased more than 1.0 log after thermophilic digestion. Overall, thermophilic anaerobic digestion performed better at reducing ARGs compared with moderate and mesophilic digestion.

### Changes in RAs of ARGs during anaerobic digestion

**Figure 2:**
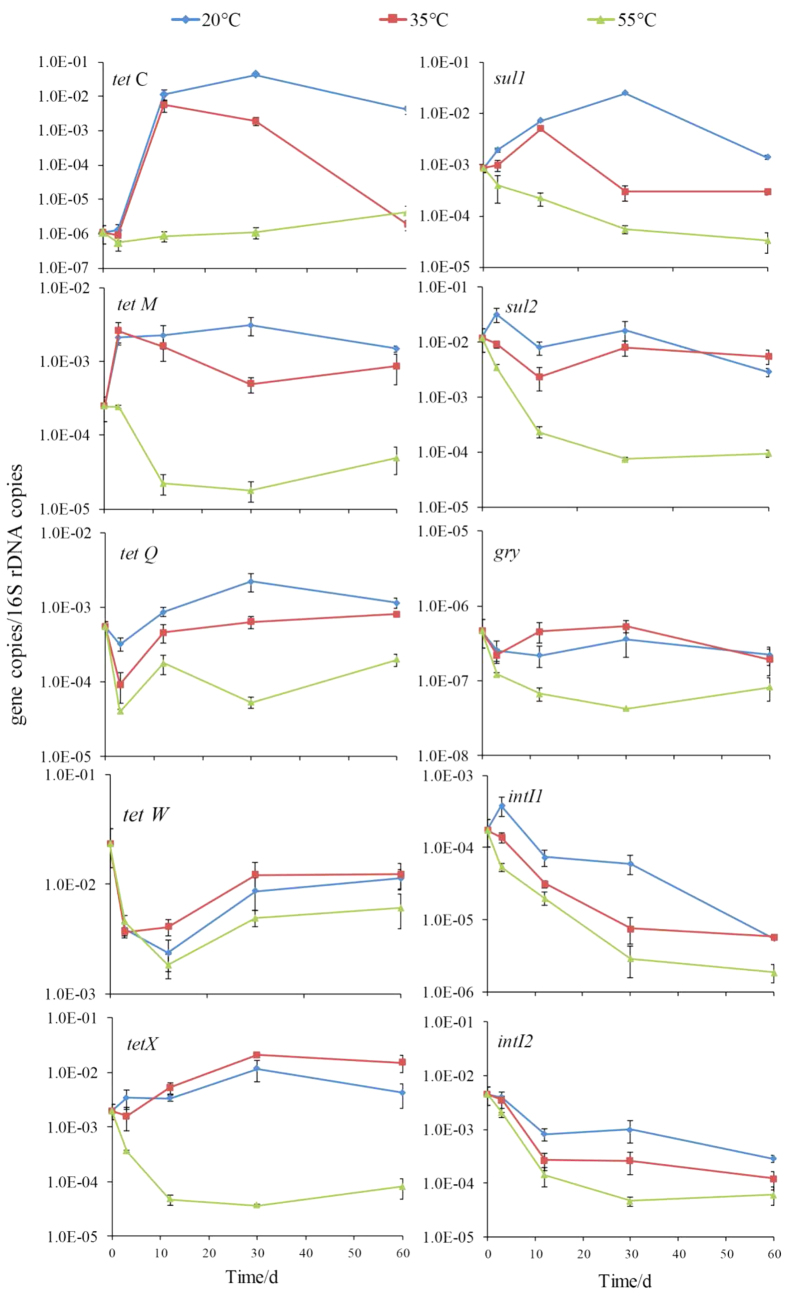
Variation in ARGs/16S rRNA gene and integrase genes/16S rRNA gene detected during anaerobic digestion at 20 °C, 35 °C, and 55 °C. Bars represent standard deviations (n = 3).

Excluding *tetC*, the RAs of all ARGs declined significantly by 1–2 orders of magnitude after thermophilic anaerobic digestion for 60 days (Fig. 2). By contrast, there were no significant declines in the RAs of all ARGs, and even significant increases in those of *tetM* and *tetX* were observed under moderate and mesophilic digestion. Similar changes in the RAs of most ARGs were found under the moderate and mesophilic treatments. However, the changes in *tetC*, *tetM*, *tetX*, and *sul1* differed under the thermophilic treatment compared with the moderate and mesophilic treatments. The variations in the RAs of *tetW*, *intI1*, and *intI2* were consistent among the three treatments. Overall, we found that *intI1* and *intI2* declined with time during anaerobic digestion, where their RAs were in the following order: thermophilic < mesophilic < moderate. Positive correlations were found between the RAs of *intI1*, *intI2*, and *sul2* (Table S3), but there were no significant correlations between the other ARGs and integrase genes.

### Bacterial community

**Figure 3:**
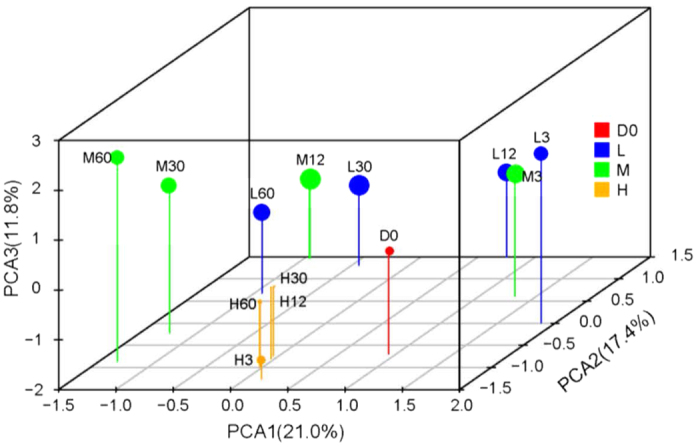
Principal components analysis based on the relative abundances of OTUs during anaerobic digestion at 20 °C (L), 35 °C (M), and 55 °C (H). D0 represents the initial material. The relative size of the circle indicates the number of OTUs.

After assembling and quality filtering, a total of 565,330 sequences were obtained, and they were clustered into 1166 OTUs at 97% similarity level. The rarefaction curve shown that the rarefied sequencing depth (33054) can reveal most bacterial communities in all samples (Figure S2). We found obvious changes in the bacterial communities in the initial digester (D0) and that obtained from the anaerobic digestion system on day 3 (Fig. 3). Three principal components (PCs) accounted for 50.2% of the total variance in bacterial community. In particular, the number of OTUs declined dramatically in the thermophilic treatment on day 3. The thermophilic treatment had the lowest OTU diversity and its bacterial community varied less with time compared with the moderate and mesophilic treatments. The bacterial communities were very similar on day 3 under the moderate and mesophilic treatments, but they started to differentiate from day 12. The trends in the bacterial community succession were similar under the moderate and mesophilic treatments, where the scores for PC1 and PC2 decreased gradually with the duration of digestion.

**Figure 4:**
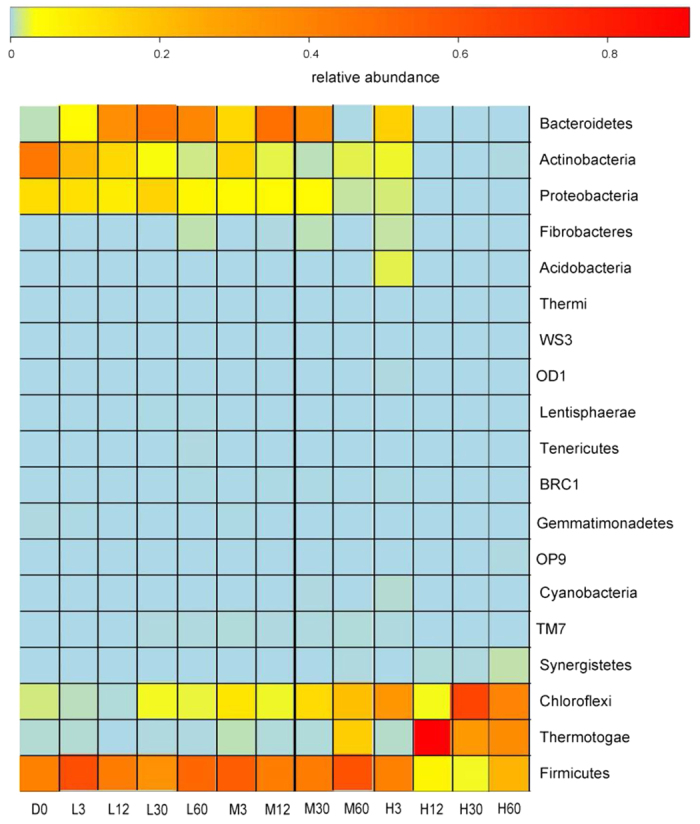
Heatmap showing the relative abundance of phyla during anaerobic digestion at 20 °C (L), 35 °C (M), and 55 °C (H). D0 represent the initial material.

Firmicutes (41.0%) and Actinobacteria (44.2%) were dominant phyla in the initial digester (Fig. 4). The phylum Firmicutes was represented primarily by the classes Bacilli and Clostridia, where Bacilli declined from 28.3% on day 0 to 0.13–0.43% on day 60, whereas Clostridia were enriched from 12.6% in the initial material to 22.5–49.7% in the digestion product. Actinobacteria primarily comprised members of the order Actinomycetales (Figure S3), which declined dramatically to 0.06–2.75% (day 60) from 44.2% (day 0) after digestion. By contrast, Bacteroidetes became dominant (41.3–50.3%) in the moderate and mesophilic systems, which was not the case under the thermophilic treatment. The phylum Chloroflexi increased with the digestion temperature, whereas Proteobacteria exhibited the opposite trend. Chloroflexi increased rapidly from 0.35% on day 0 to 31.7% and 38.8% on day 60 under the mesophilic and thermophilic treatments, respectively, but only 1.5% under the moderate treatment. Thermotogae and Chloroflexi were dominant phyla in the thermophilic treatment, where they accounted for 76.1–97.0% from day 12 to 60. Thermotogae mainly comprised genus S1 and Chloroflexi were represented primarily by an unnamed genus belonging to family SHA-31 (Figure S4).

### Relationships between ARGs, bacterial communities, and environmental factors

There were significant positive correlations between Actinobacteria and integrase genes (Table 1). Bacteroidetes had significant positive correlations with six ARGs (*tetC*, *tetM*, *tetQ*, *tetX*, *sul1*, and *gyrA*). Proteobacteria had significant positive correlations with all ARGs and integrase genes, except for *tetW* and *tetX*. There were no significant correlations between Firmicutes and ARG or integrase genes, although class Bacilli in Firmicutes had significant positive correlations with *sul2*, *intI1*, and *intI2* (Table S4). Chloroflexi and Thermotogae had negative correlations with all ARGs and integrase genes, but significant correlations were only found between Thermotogae and *tetM* and *gyrA*.

**Figure 5:**
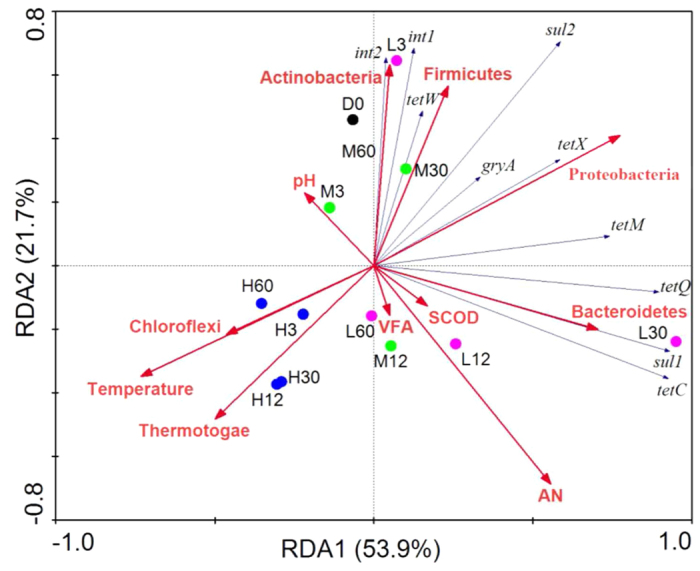
Redundancy analysis to assess the relationships among environmental factors (red arrows), main bacterial phyla (red arrows), and ARGs (blue arrows). L represents moderate treatment (20 °C), M represents mesophilic treatment (35 °C), and H represents thermophilic treatment (55 °C). AN: available nitrogen; VFA: volatile fatty acids; SCOD: soluble chemical oxygen demand.

Redundancy analysis (RDA) was used to assess the relationships among environmental factors, bacterial communities and ARGs. The bacterial community and selected environmental factors explained 75.6% of the total variance in the ARGs. Among the selected variables, six phyla explained the most variation, i.e., 60.4%, while temperature explained 16.9%, AN explained 14.5% and the other three factors only accounted for 8.2% of the total variance. There were significant negative correlations between temperature and Proteobacteria, Actinobacteria, Bacteroidetes and Firmicutes (Table S5). Chloroflexi and Thermotogae had significant positive correlations with temperature while Bacteroidetes had significant positive correlation with AN. The ARG profiles in the initial material were similar to those in the early stage for the moderate system (3 days) and the later stages for the mesophilic system (30 and 60 days) (Fig. 5). The ARGs profiles varied greatly with the digestion time under moderate and mesophilic digestion, whereas they varied less in the thermophilic system, although they were significantly different from that in the initial material.

## Discussion

In this study, we detected diverse and abundant ARGs in dairy manure, which was consistent with previous studies^7^,^28^,^29^, thereby indicating the importance of animal manure as a reservoir for ARGs. However, the types and abundances of the 20 assessed ARGs differed greatly from those found in wastewater sludge^19^,^30^,^31^,^32^. In general, many different types of ARGs can be found in wastewater sludge, but the most common ARG types observed in animal manure are tetracycline resistance genes and sulfonamide resistance genes^30^,^31^,^32^. This is attributable mainly to the different antibiotic contact histories of the two environments^4^,^21^,^22^.

Similar to previous studies^17^,^33^, the present results also found the thermophilic anaerobic digestion performed better at declining ARGs than moderate and mesophilic digestion. However, Diehl and Lapara^17^ showed that mesophilic anaerobic digestion removed most of the *tet* genes, whereas an enrichment effect was obtained under mesophilic digestion in the present study. The decline in *tet* genes after mesophilic anaerobic digestion was also observed by Zhang *et al*., but they found no difference in the removal of *tet* genes under mesophilic and thermophilic condition^20^. Significant reduction of *sul1* and *sul2* by mesophilic and thermophilic digestion was consistent to Ma *et al*.^19^ and Miller *et al*.^34^. By contrast, Zhang *et al*. showed that *sul1* and *sul2* were enriched under both mesophilic and thermophilic digestion^20^. The inconsistent results may relate to the different characteristics of the raw materials tested (dairy manure in this study, mixture of primary and secondary solids^17^,^19^, concentrated aerobic sludge^20^) and the diverse operating conditions (TS, VS, material retention time, stirring rate etc.).

The RAs of ARGs could reflect the ratio of antibiotic resistance bacteria relative to the total bacteria, and changes in the RAs of ARGs might be attributable to variations in their host bacteria ratio as well as the horizontal gene transfer (HGT) of ARGs among bacteria^15^,^35^. The low RAs of most ARGs in the thermophilic reactor throughout digestion suggest that high temperature can reduce the ratio of antibiotic-resistant bacteria. For most of the ARGs and integrase genes, the changes in their RAs occurred mostly within 30 days. This is because the bacterial communities varied greatly for 30 days but they tended to stabilize in the later stage^36^. The similar changes in the ARGs under the moderate and mesophilic treatments may be explained by their similar bacterial communities during digestion. By contrast, higher temperature digestion (55 °C) led to remarkable differences in the bacterial community compared with those obtained at 20 °C and 35 °C^18^,^37^, where different trends in *tetC*, *tetM*, and *tetX* were observed under the thermophilic, moderate, and mesophilic treatments. Integrons are often associated with mobile genetic elements such as transposons and plasmids, and they are recognized as important indicators of HGT^35^,^38^,^39^. It is considered that HGT may play important roles in anaerobic digestion process because high biomass densities may provide favorable conditions for HGT^40^. However, we found no significant correlation between ARGs (except *sul2*) and integrase genes, which may suggest that the integrons do not play a large role in potential HGT during anaerobic digestion^41^.

Very high variations in the oxygen concentration and temperature were the main reasons for the changes in the bacterial communities^18^,^37^. Most of the mesophilic bacteria were eliminated at 55 °C, and thus this treatment had the lowest bacterial diversity^18^,^37^. The dramatic decline in Actinobacteria after digestion was attributable mainly to decreases in the order Actinomycetales (Figure S3), which are mostly aerobic^42^. The order Bacteroidales was dominant in the phylum Bacteroidetes and they are obligate anaerobes but not thermotolerant^43^, and thus they increased in the 20 °C and 35 °C treatments, whereas they were not found in the 55 °C treatment. Two classes of Firmicutes exhibited differences because Clostridia are anaerobic whereas Bacilli are obligate or facultative aerobes^44^. Chloroflexi and Thermotogae are anaerobic thermophilic bacteria^37^, and they were only found in the 35 °C and 55 °C treatments. It has been reported that Chloroflexi can use halogenated organics as energy sources^45^ and that Thermotogae can utilize different complex carbohydrates to produce hydrogen gas during anaerobic digestion^46^.

This study confirmed that changes in the bacterial communities are key drivers of changes in the variation of ARGs during anaerobic digestion. Similar results were obtained based on tests of aerobic composting and water chlorination processes^41^,^47^. Miller *et al*.^48^ also found that the ARG level of a digester was attributed to the survival or death of antibiotic resistant bacteria during anaerobic digestion. The variations in six phyla (Actinobacteria, Bacteroidetes, Chloroflexi, Firmicutes, Proteobacteria, and Thermotogae) were the main causes of the changes during anaerobic digestion and the differences at various temperatures. By contrast, the chemical characteristics of the digestion system were not correlated with the changes in ARGs. The results of the present study indicate that Proteobacteria and Bacteroidetes are important hosts of ARGs during anaerobic digestion (Table 1), which is consistent with previous reports that Bacteroidetes and Proteobacteria can resist a wide variety of antibiotics, including tetracycline, β-lactams, aminoglycosides, and erythromycin^49^,^50^. The high temperature treatment effectively eliminated Proteobacteria and Bacteroidetes, thereby reducing *tetM*, *tetQ*, *gyrA*, and *sul1* with high efficiency. Similarly, the increase in Bacteroidetes was responsible for the increases of *tetC*, *tetM*, *tetQ*, *tetX*, and *sul1* under moderate and mesophilic temperatures during anaerobic digestion. In addition, the inhibition of HGT at high temperatures may also contribute to the lower RAs of ARGs in the thermophilic system^48^,^51^.

Anaerobic digestion can remove some pathogens from raw waste and thermophilic digestion is particularly effective^52^,^53^. Several potentially opportunistic human pathogens such as Bacilli and Actinomycetales were removed by anaerobic digestion in this study. However, the abundance of Bacteroidetes, which includes some species that can cause infections of the peritoneal cavity, gastrointestinal surgery, and appendicitis^54^, increased after moderate and mesophilic digestion. Actinomycetales and *Corynebacterium* are potential pathogens that can harbor *sul* and integrons^7^,^55^, where they comprised 29.1% of the total bacteria in the raw material, but they were removed by thermophilic digestion, whereas 0.26% and 0.17% remained under moderate and mesophilic digestion. Proteobacteria are also potentially pathogenic bacteria^56^, which were removed by thermophilic digestion, but 5.5% and 1.7% remained after moderate and mesophilic digestion. These results suggest that the digestion products from moderate and mesophilic treatments are potential risks for agricultural application^15^,^52^.

Interestingly, in agreement with the results of most previous studies^12^,^13^,^14^,^17^,^19^,^34^, integrase genes were significantly reduced at different temperatures, thereby demonstrating the universal reduction effect of anaerobic digestion on integrase genes. Proteobacteria, order Actinomycetales in Actinobacteria, and class Bacilli in Firmicutes were the main carriers of integrons in the present study. Actinomycetales were the main producer of antibiotics, and thus they were common hosts of ARGs and integrons^57^,^58^. Proteobacteria and Bacilli have also been reported as common carriers of integrons^59^,^60^. The universal reduction of integrons by anaerobic digestion can be explained by the significant reduction of aerobic bacteria such as Actinomycetales and Bacilli after anaerobic digestion of the raw material.

In conclusion, the present study demonstrated that bacterial community succession was the main mechanism that affected the variation in ARGs and integrase genes. Thermophilic anaerobic digestion reduced the abundance of mesophilic bacteria carrying ARGs and its ARG reduction was better than that of the moderate and mesophilic treatments. Anaerobic digestion universally reduced the abundance of integrase genes because it removed significant amounts of the aerobic hosts of integrons. Thermophilic digestion also has advantages in terms of methane production as well as speed and stability compared with moderate and mesophilic digestion^33^. The consumption of more energy to maintain a high temperature is the main disadvantage of thermophilic anaerobic digestion. However, given the reduction of ARGs, integrase genes, and pathogenic bacteria, as well as methane production, thermophilic anaerobic digestion is highly recommended for the treatment and reuse of animal manure.

## Additional Information

**How to cite this article**: Sun, W. *et al*. Mechanism and Effect of Temperature on Variations in Antibiotic Resistance Genes during Anaerobic Digestion of Dairy Manure. *Sci. Rep.* **6**, 30237; doi: 10.1038/srep30237 (2016).

## Supplementary Material

Supplementary Information

## Figures and Tables

**Table 1:** Pearson’s correlation coefficients between phyla with relative abundance >1.0% and ARGs and integrase genes.

Phylum	*tetC*	*tetM*	*tetQ*	*tetW*	*tetX*	*sul1*	*sul2*	*gryA*	*int1*	*int2*
Actinobacteria	−0.127	0.124	−0.060	0.385	−0.215	−0.068	0.544	0.370	0.703**	0.865**
Bacteroidetes	0.584*	0.604*	0.695**	0.300	0.562*	0.569*	0.027	0.599*	−0.259	−0.354
Chloroflexi	−0.287	−0.529	−0.370	−0.192	−0.101	−0.322	−0.448	−0.541	−0.409	−0.386
Firmicutes	−0.029	0.482	0.101	0.150	0.180	0.007	0.520	0.393	0.505	0.553
Proteobacteria	0.601*	0.668*	0.634*	0.306	0.199	0.642*	0.782**	0.605*	0.613*	0.568*
Thermotogae	−0.237	−0.558*	−0.400	−0.437	−0.431	−0.258	−0.447	−0.604*	−0.292	−0.342

*Significant at *P* < 0.05, **Significant at *P* < 0.01.

## References

[b1] World Health Organization: Global strategy for the containment of antimicrobial resistance. URL: http://www.who.International/emc/diseases/zoo/edg/draft.html (2004).

[b2] Ma L., Li B. & Zhang T. Abundant rifampin resistance genes and significant correlations of antibiotic resistance genes and plasmids in various environments revealed by metagenomic analysis. Appl. Microbiol. Biotechnol. 98, 5195–5204 (2014). 24615381 10.1007/s00253-014-5511-3

[b3] Pruden A., Pei R., Storteboom H. & Carlson K. H. Antibiotic resistance genes as emerging contaminants: studies in Northern Colorado. Environ. Sci. Technol. 40, 7445–7450 (2006). 17181002 10.1021/es060413l

[b4] Zhou L. J. . Use patterns, excretion masses and contamination profiles of antibiotics in a typical swine farm, south China. Environ. Sci.-Process Impacts 15, 802–813 (2013). 23411720 10.1039/c3em30682h

[b5] Durso L. M. & Cook K. L. Impacts of antibiotic use in agriculture: what are the benefits and risks? Curr. Opin. Microbiol. 19, 37–44 (2014). 24997398 10.1016/j.mib.2014.05.019

[b6] Zhao L., Dong Y. H. & Wang H. Residues of veterinary antibiotics in manures from feedlot livestock in eight provinces of China. Sci. Total Environ. 408, 1069–1075 (2010). 19954821 10.1016/j.scitotenv.2009.11.014

[b7] Yang Q. X. . Distribution of antibiotic-resistant bacteria in chicken manure and manure-fertilized vegetables. Environ. Sci. Pollut. Res. 21, 1231–1241 (2014). 10.1007/s11356-013-1994-1 23892601

[b8] Heuer H., Schmitt H. & Smalla K. Antibiotic resistance gene spread due to manure application on agricultural fields. Curr. Opin. Microbiol. 14, 236–243 (2011). 21546307 10.1016/j.mib.2011.04.009

[b9] Pruden A., Arabi M. & Storteboom H. N. Correlation between upstream human activities and riverine antibiotic resistance genes. Environ. Sci. Technol. 46, 11541–11549 (2012). 23035771 10.1021/es302657r

[b10] Pruden A. . Management options for reducing the release of antibiotics and antibiotic resistance genes to the environment. Environ. Health Perspect. 121, 878–885 (2013). 23735422 10.1289/ehp.1206446 PMC3734499

[b11] Wang L. L. . Persistence of resistance to erythromycin and tetracycline in swine manure during simulated composting and lagoon treatments. Microb. Ecol. 63, 32–40 (2012). 21811793 10.1007/s00248-011-9921-9

[b12] Joy S. R. . Fate of antimicrobials and antimicrobial resistance genes in simulated swine manure storage. Sci. Total Environ. 481, 69–74 (2014). 24583946 10.1016/j.scitotenv.2014.02.027

[b13] Ghosh S., Ramsden S. J. & LaPara T. M. The role of anaerobic digestion in controlling the release of tetracycline resistance genes and class 1 integrons from municipal wastewater treatment plants. Appl. Microbiol. Biotechnol. 84, 791–796 (2009). 19597810 10.1007/s00253-009-2125-2

[b14] Beneragama N. . The survival of cefazolin-resistant bacteria in mesophilic co-digestion of dairy manure and waste milk. Waste Manage. Res. 31, 843–848 (2013). 10.1177/0734242X13477717 23512952

[b15] Resende J. A. . Dynamics of antibiotic resistance genes and presence of putative pathogens during ambient temperature anaerobic digestion. J. Appl. Microbiol. 117, 1689–1699 (2014). 25250562 10.1111/jam.12653

[b16] Chae K. J., Jang A., Yim S. K. & Kim I. S. The effects of digestion temperature and temperature shock on the biogas yields from the mesophilic anaerobic digestion of swine manure. Bioresour. Technol. 99, 1–6 (2008). 17306978 10.1016/j.biortech.2006.11.063

[b17] Diehl D. L. & LaPara T. M. Effect of temperature on the fate of genes encoding tetracycline resistance and the integrase of class 1 integrons within anaerobic and aerobic digesters treating municipal wastewater solids. Environ. Sci. Technol. 44, 9128–9133 (2010). 21058743 10.1021/es102765a

[b18] Cho H. U., Kim Y. M., Choi Y., Kim H. G. & Park J. M. Influence of temperature on volatile fatty acid production and microbial community structure during anaerobic fermentation of microalgae. Bioresour. Technol. 191, 475–480 (2015). 25791331 10.1016/j.biortech.2015.03.009

[b19] Ma Y. J. . Effect of various sludge digestion conditions on sulfonamide, macrolide, and tetracycline resistance genes and class I integrons. Environ. Sci. Technol. 45, 7855–7861 (2011). 21815642 10.1021/es200827t

[b20] Zhang T., Yang Y. & Pruden A. Effect of temperature on removal of antibiotic resistance genes by anaerobic digestion of activated sludge revealed by metagenomic approach. Appl. Microbiol. Biotechnol. 99, 7771–7779 (2015). 25994259 10.1007/s00253-015-6688-9

[b21] Stasinakis A. S. Review on the fate of emerging contaminants during sludge anaerobic digestion. Bioresour. Technol. 121, 432–440 (2012). 22853968 10.1016/j.biortech.2012.06.074

[b22] An J., Chen H. W., Wei S. H. & Gu J. Antibiotic contamination in animal manure, soil, and sewage sludge in Shenyang, northeast China. Environ. Earth Sci. 74, 5077–5086 (2015).

[b23] Aydin S., Shahi A., Ozbayram E. G., Ince B. & Ince O. Use of PCR-DGGE based molecular methods to assessment of microbial diversity during anaerobic treatment of antibiotic combinations. Bioresour. Technol. 192, 735–740 (2015). 26101963 10.1016/j.biortech.2015.05.086

[b24] Tian Z., Zhang Y., Li Y., Chi Y. & Yang M. Rapid establishment of thermophilic anaerobic microbial community during the one-step startup of thermophilic anaerobic digestion from a mesophilic digester. Water Res. 69, 9–19 (2015). 25463927 10.1016/j.watres.2014.11.001

[b25] Edgar R. C. UPARSE: highly accurate OTU sequences from microbial amplicon reads. Nat. Methods 10, 996–998 (2013). 23955772 10.1038/nmeth.2604

[b26] Edgar R. C. Search and clustering orders of magnitude faster than BLAST. Bioinformatics 26, 2460–2461 (2010). 20709691 10.1093/bioinformatics/btq461

[b27] Maidak B. L. . The RDP (ribosomal database project). Nucleic Acids Res. 25, 109–110 (1997). 9016515 10.1093/nar/25.1.109 PMC146422

[b28] Zhu Y. G. . Diverse and abundant antibiotic resistance genes in Chinese swine farms. P. Natl. Acad. Sci. USA 110, 3435–3440 (2013). 10.1073/pnas.1222743110 PMC3587239 23401528

[b29] Wichmann F., Udikovic-Kolic N., Andrew S. & Handelsman J. Diverse antibiotic resistance genes in dairy cow manure. mbio 5, 379–382 (2014). 10.1128/mBio.01017-13 PMC3993861 24757214

[b30] Zhang T., Zhang X. X. & Ye L. Plasmid metagenome reveals high levels of antibiotic resistance genes and mobile genetic elements in activated sludge. PLoS One 6, e26041 (2011). 22016806 10.1371/journal.pone.0026041 PMC3189950

[b31] Karkman A. . High-throughput quantification of antibiotic resistance genes from an urban wastewater treatment plant. FEMS Microbial. Ecol. fiw014 (2016). 10.1093/femsec/fiw014 26832203

[b32] Baker-Austin C., Wright M. S., Stepanauskas R. & McArthur J. V. Co-selection of antibiotic and metal resistance. Trends Microbiol. 14, 176–182 (2006). 16537105 10.1016/j.tim.2006.02.006

[b33] Beneragama N. . The survival of multidrug-resistant bacteria in thermophilic and mesophilic anaerobic co-digestion of dairy manure and waste milk. Anim. Sci. J. 84, 426–433 (2013). 23607603 10.1111/asj.12017

[b34] Miller J. H. . Effect of Silver Nanoparticles and Antibiotics on Antibiotic Resistance Genes in Anaerobic Digestion. Water Environ. Res. 85, 411–421 (2013). 23789571 10.2175/106143012x13373575831394

[b35] Thomas C. M. & Nielsen K. M. Mechanisms of, and barriers to, horizontal gene transfer between bacteria. Nat. Rev. Microbiol. 3, 711–721 (2005). 16138099 10.1038/nrmicro1234

[b36] Yan Z. . The effects of initial substrate concentration, C/N ratio, and temperature on solid-state anaerobic digestion from composting rice straw. Bioresour. Technol. 177, 266–273 (2015). 25496947 10.1016/j.biortech.2014.11.089

[b37] Pervin H. M. . Drivers of microbial community composition in mesophilic and thermophilic temperature-phased anaerobic digestion pre-treatment reactors. Water Res. 47, 7098–7108 (2013). 24216229 10.1016/j.watres.2013.07.053

[b38] Fluit A. C. & Schmitz F. J. Class 1 integrons, gene cassettes, mobility, and epidemiology. Eur. J. Clin. Microbiol. Infect. Dis. 18, 761–770 (1999). 10614949 10.1007/s100960050398

[b39] Chen B. . The role of class I integrons in the dissemination of sulfonamide resistance genes in the Pearl River and Pearl River Estuary, South China. J. Hazard. Mater. 282, 61–67 (2015). 24994022 10.1016/j.jhazmat.2014.06.010

[b40] Snyder L. & Champness W. Molecular genetics of bacteria, 3rd edn; ASM Press: Washington, D.C. 2007.

[b41] Su J. . Antibiotic resistome and its association with bacterial communities during sewage sludge composting. Environ. Sci. Technol. 49, 7356–7363 (2015). 26018772 10.1021/acs.est.5b01012

[b42] Öner Ö. . Cultivable sponge-associated actinobacteria from coastal area of Eastern Mediterranean Sea. Advances in Microbiology 4, 306–316 (2014).

[b43] Li Y. F. . Comparison of the microbial communities in solid-state anaerobic digestion (SS-AD) reactors operated at mesophilic and thermophilic temperatures. Appl. Microbiol. Biotechnol. 99, 969–980 (2015). 25194839 10.1007/s00253-014-6036-5

[b44] Chang J. J. . Syntrophic co-culture of aerobic Bacillus and anaerobic Clostridium for bio-fuels and bio-hydrogen production. Int. J. Hydrogen Energ. 33, 5137–5146 (2008).

[b45] Krzmarzick M. J. . Natural niche for organohalide-respiring Chloroflexi. Appl. Environ. Microbiol. 78, 393–401 (2012). 22101035 10.1128/AEM.06510-11 PMC3255752

[b46] Nguyen T. A. D., Kim J. P., Kim M. S., Oh Y. K. & Sim S. J. Optimization of hydrogen production by hyperthermophilic eubacteria, Thermotoga maritima and Thermotoga neapolitana in batch fermentation. Int. J. Hydrogen Energ 33, 1483–1488 (2008).

[b47] Jia S. . Bacterial community shift drives antibiotic resistance promotion during drinking water chlorination. Environ. Sci. Technol. 49, 12271–12279 (2015). 26397118 10.1021/acs.est.5b03521

[b48] Miller J. H., Novak J. T., Knocke W. R. & Pruden A. Survival of antibiotic resistant bacteria and horizontal gene transfer control antibiotic resistance gene content in anaerobic digesters. Front. Microbiol. 7 (2016). 10.3389/fmicb.2016.00263 PMC4781833 27014196

[b49] Rampelli S. . Metagenome sequencing of the Hadza hunter-gatherer gut microbiota. Curr. Biol. 25, 1682–1693 (2015). 25981789 10.1016/j.cub.2015.04.055

[b50] Hu Y. . Metagenome-wide analysis of antibiotic resistance genes in a large cohort of human gut microbiota. Nat. Commun. 4, 2151 (2013). 23877117 10.1038/ncomms3151

[b51] Guan J., Wasty A., Grenier C. & Chan M. Influence of temperature on survival and conjugative transfer of multiple anti biotic-resistant Plasmids in chicken manure and compost microcosms. Poult. Sci. 86, 610–613 (2007). 17369529 10.1093/ps/86.4.610

[b52] Resende J. A. . Prevalence and persistence of potentially pathogenic and antibiotic resistant bacteria during anaerobic digestion treatment of cattle manure. Bioresour. Technol. 153, 284–291 (2014). 24374028 10.1016/j.biortech.2013.12.007

[b53] Sahlstrom L. A review of survival of pathogenic bacteria in organic waste used in biogas plants. Bioresour. Technol. 87, 161–166 (2003). 12765355 10.1016/s0960-8524(02)00168-2

[b54] Wexler H. M. Bacteroides: the good, the bad, and the nitty-gretty. Clin. Microbiol. Rev. 20, 593–621 (2007). 17934076 10.1128/CMR.00008-07 PMC2176045

[b55] Nešvera J., Hochmannová J. & Pátek M. An integron of class 1 is present on the plasmid pCG4 from gram-positive bacterium Corynebacterium glutamicum. FEMS Microbiol. Lett. 169, 391–395 (1998). 9868786 10.1111/j.1574-6968.1998.tb13345.x

[b56] Boucher H. W. . Bad bugs, no drugs: No ESKAPE! An update from the infectious diseases society of America. Clin. Infect. Dis. 48, 1–12 (2009). 19035777 10.1086/595011

[b57] D ’Costa V. M., McGrann K. M., Hughes D. W. & Wright G. D. Sampling the Antibiotic Resistome. Science 311, 374–377 (2006). 16424339 10.1126/science.1120800

[b58] Stegmann E., Frasch H. J., Kilian R. & Pozzi R. Self-resistance mechanisms of actinomycetes producing lipid II-targeting antibiotics. Int. J. Med. Microbiol. 305, 190–195 (2014). 25601631 10.1016/j.ijmm.2014.12.015

[b59] Mazel D., Dychinco B., Webb V. A. & Davies J. Antibiotic resistance in the ECOR collection: integrons and identification of a novel aad gene. Antimicrob. Agents Chemother. 44, 1568–1574 (2000). 10817710 10.1128/aac.44.6.1568-1574.2000 PMC89914

[b60] Martinez E., Djordjevic S., Stokes H. W. & Chowdhury P. R. Mobilized Integrons: Team Players in the Spread of Antibiotic Resistance Genes//Lateral Gene Transfer in Evolution. Springer: New York, 79–103 (2013).

