# Supplementary material for: Mechanism and Effect of Temperature on Variations in Antibiotic Resistance Genes during Anaerobic Digestion of Dairy Manure
Source: Sci Rep. 2016 Jul 22;6:30237. doi: 10.1038/srep30237 (PMC4957233; doi:10.1038/srep30237)
Supplement: Supplementary Information [file srep30237-s1.pdf]

Supplementary Information

Title: Mechanism and Effect of Temperature on Variations in Antibiotic Resistance Genes during Anaerobic Digestion of Dairy Manure

Authors: Wei Sun, Xun Qian, Jie Gu*, Xiao-Juan Wang, and Man-Li Duan
*Correspondence author, email: gujie205@sina.com

Address: College of Natural Resources and Environment, Northwest A&F University, Yangling, Shaanxi 712100, China

Table S1 Characteristics of dairy manure and the inoculum

	TS (%)	pH	Total C (g/kg)	Total N (g/kg)
dairy manure	91.3%	8.54	413.0	16.2
inoculum	8.8%	7.52	392.4	21.2

Table S2 PCR primers used in this study

Gene Name	Forward Primer	Reverse Primer	Annealing Temperature	References
*tet*A	GCGCGATCTGGTTCACTCG	AGTCGACAGYRGCGCCGGC	54°C	1
*tet*B	AAAACTTATTATATTATAGTC	TGGAGTATCAATAATATTCAC	46°C	2
*tet*C	GCGGGATATCGTCCATTCCG	GCGTAGAGGATCCACAGGACG	59°C	1
*tet*E	GTTATTACGGGAGTTTGTTGG	AATACAACACCCACACTACGC	54°C	1
*tet*G	GCAGAGCAGGTCGCTGG	CCYGCAAGAGAAGCCAGAAG	54°C	1
*tet*M	ACAGAAAGCTTATTATATAAC	TGGCGTGTCTATGATGTTCAC	52°C	2
*tet*O	ATGTGGATACTACAACGCATGAGATT	TGCCTCCACATGATATTTTTCCT	48°C	2
*tet*Q	AGAATCTGCTGTTTGCCAGTG	CGGAGTGTCAATGATATTGCA	55°C	2
*tet*T	AAGGTTTATTATATAAAAGTG	AGGTGTATCTATGATATTTAC	46°C	2
*tet*W	GAGAGCCTGCTATATGCCAGC	GGGCGTATCCACAATGTTAAC	56°C	2
*tet*X	CAATAATTGGTGGTGGACCC	TTCTTACCTTGGACATCCCG	55°C	3
*sul*1	CGGCGTGGGCTACCTGAACG	GCCGATCGCGTGAAGTTCCG	60°C	4
*sul*2	GCGCTCAAGGCAGATGGCATT	GCGTTTGATACCGGCACCCGT	59°C	4
*sul*A	TCTTGAGCAAGCACTCCAGCAG	TCCAGCCTTAGCAACCACATGG	57°C	4
*dfr*A1	AGCATTACCCAACCGAAAGT	TGTCAGCAAGATAGCCAGAT	60°C	4
*dfr*A7	AAATGGCGTAATCGGTAATG	GTGAACAGTAGACAAATGAAT	51°C	4
*gry*A	CGATGTCGGTCATTGTTGGC	ATACCTACGGCGATACCGGA	61°C	5
*par*C	GCCTAAACAACGCACGGAAA	TGACACGGGAGGTAACCAGA	53°C	5
*qnr*C	TTCGATCGGACTGCTTGTGG	AACACATGGTGCAGGGGATT	53°C	5
*qnr*S	CCCCATGCCCGAAGTTATCA	ACTGCTTGGAGTGTGTTGGT	53°C	5
*intI*1	CTGGATTTCGATCACGGCACG	ACATGCGTGTAAATCATCGTCG	60°C	4
*intI*2	GTTATTTTATTGCTGGGATTAGGC	TTTTACGCTGCTGTATGGTGC	55°C	6
16S rRNA	CCTACGGGAGGCAGCAG	ATTACCGCGGCTGCTGG	55°C	1

Table S3 Pearson's correlation coefficients between the relative abundances of ARGs, integrase genes

	tetM	*tetQ*	*tetW*	*tetX*	*sul1*	*sul2*	*gryA*	*int1*	*int2*
tetC	**0.635***	**0.877****	–0.003	0.305	**0.992****	0.256	0.267	–0.082	–0.133
tetM	1	**0.583***	–0.128	0.176	**0.679***	**0.553***	0.288	0.373	0.248
tetQ		1	0.224	0.518	**0.845****	0.259	0.397	–0.122	–0.178
tetW			1	0.325	0.005	–0.032	**0.722****	–0.064	0.133
tetX				1	0.264	0.168	**0.607***	–0.204	–0.279
sul1					1	0.317	0.282	0.003	–0.065
sul2						1	0.344	**0.895****	**0.665***
gryA							1	0.172	0.244
int1								1	**0.837****
int2									1

*significant at $P < 0.05$, **significant at $P < 0.01$

Table S4 Pearson's correlation coefficients between representative bacterial communities (assigned to the lowest level that accounted for >90% in each phylum) and ARGs

Representative phylum/class/order/family/genus	*tetC*	*tetM*	*tetQ*	*tetW*	*tetX*	*sul1*	*sul2*	*gryA*	*int1*	*int2*
p_Chloroflexi; c_Anaerolineae; o_SBR1031;f_SHA-31; g_Unnamed	–0.299	–0.541	–0.379	–0.195	–0.100	–0.334	–0.452	–0.546	–0.411	–0.398
p_Bacteroidetes; c_Bacteroidia; o_Bacteroidales	**0.583***	**0.591***	**0.694****	0.301	**0.564***	**0.566***	0.006	**0.592***	–0.282	–0.371
p_Firmicutes; c_Bacilli	–0.172	0.162	–0.125	0.258	–0.228	–0.102	**0.721****	0.307	**0.877****	**0.901****
p_Firmicutes; c_Clostridia	0.067	0.416	0.181	0.014	0.324	0.066	0.140	0.243	0.033	0.068
p_Thermotogae; c_Thermotogae; o_Thermotogales; f_Thermotogaceae; g_S1	–0.237	**–0.558***	–0.400	–0.437	–0.431	–0.258	–0.447	**–0.604***	–0.292	–0.342
p_Proteobacteria	**0.601***	**0.668***	**0.634***	0.306	0.199	**0.642***	**0.782****	**0.605***	**0.613***	**0.568***
p_Actinobacteria; c_Acidimicrobiia; o_Acidimicrobiales	–0.128	0.123	–0.061	0.385	–0.215	–0.069	0.544	0.369	**0.703****	**0.865****

*significant at $P < 0.05$, **significant at $P < 0.01$

Table S5 Pearson's correlation coefficients between bacterial communities and environmental factors

	Proteobacteria	Actinobacteria	Bacteroidetes	Chloroflexi	Thermotogae	Firmicutes
pH	–0.092	0.179	–0.190	0.224	0.141	–0.355
SCOD	0.322	0.140	0.256	–0.211	–0.130	0.001
AN	0.124	–0.551	**0.704****	–0.173	–0.057	–0.109
VFA	0.012	0.016	0.030	–0.236	–0.228	0.561
Temperature	**–0.792****	–0.326	**–0.665***	**0.637***	**0.707****	**–0.558***

AN: available nitrogen; VFA: volatile fatty acids; SCOD: soluble chemical oxygen demand.

*significant at $P < 0.05$, **significant at $P < 0.01$

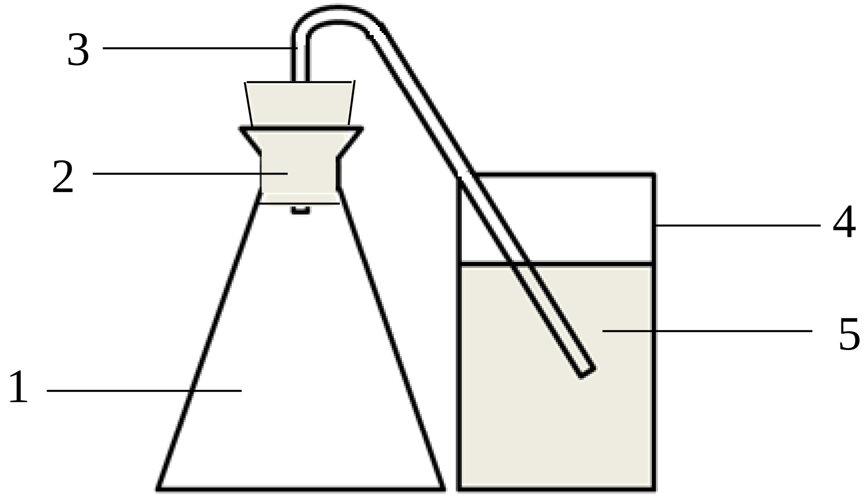

Figure S1 Diagrammatic sketch of the digestion reactor. 1: triangular flask, 2: rubber plug, 3: rubber hose, 4: assistant bottle, 5: water.

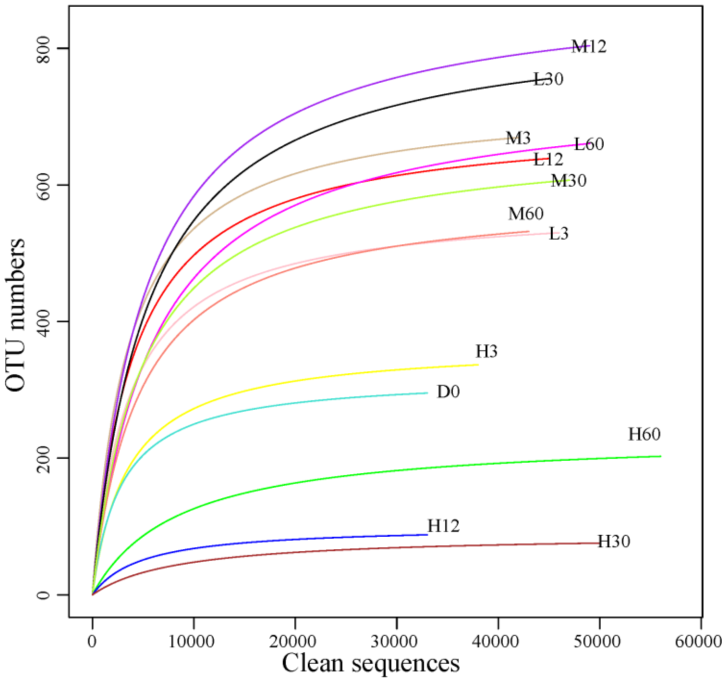

Figure S2 Rarefaction curves of sequenced samples. L represents moderate treatment (20°C), M represents mesophilic treatment (35°C), and H represents thermophilic treatment (55°C).

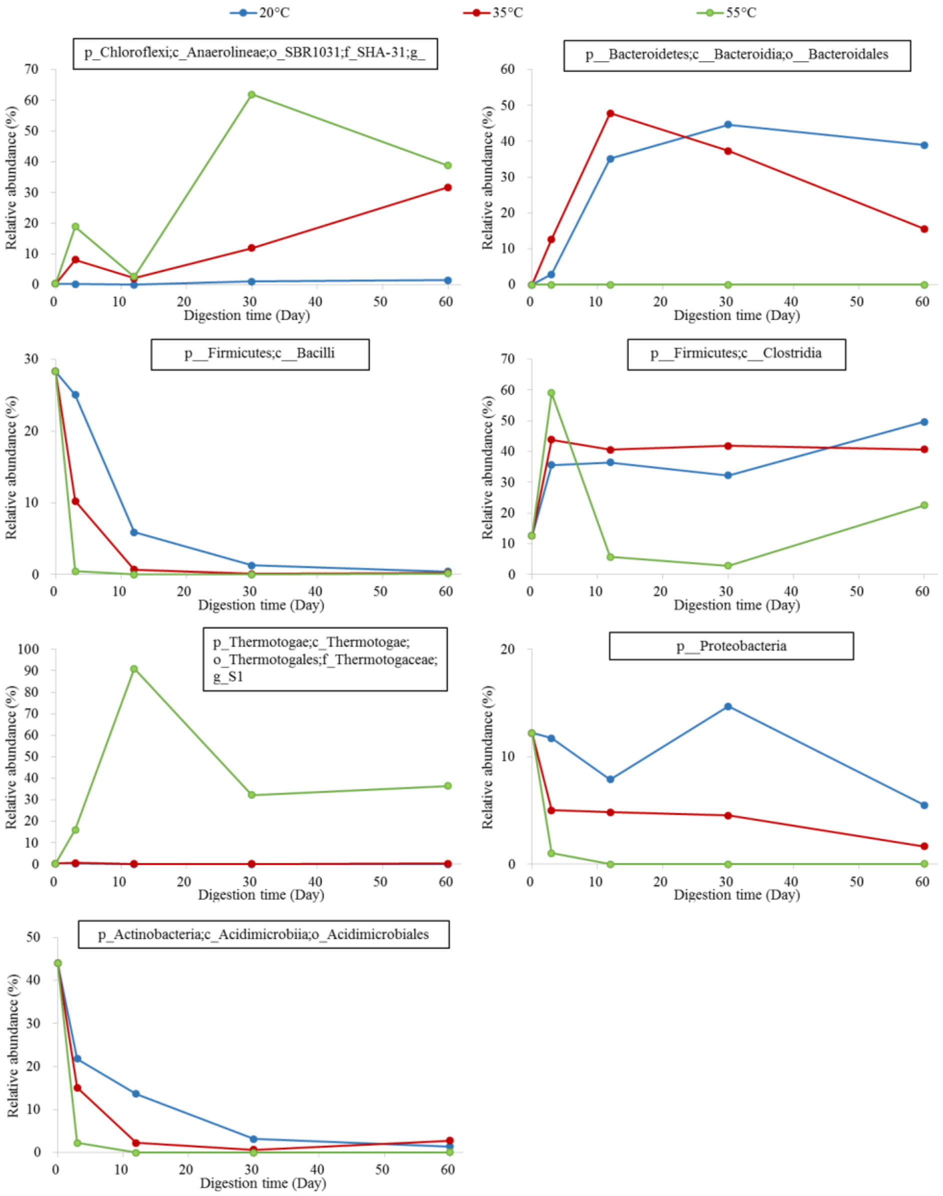

Figure S3 Variation in the bacterial communities assigned to the lowest level that accounted for >90% in each phylum during anaerobic digestion.

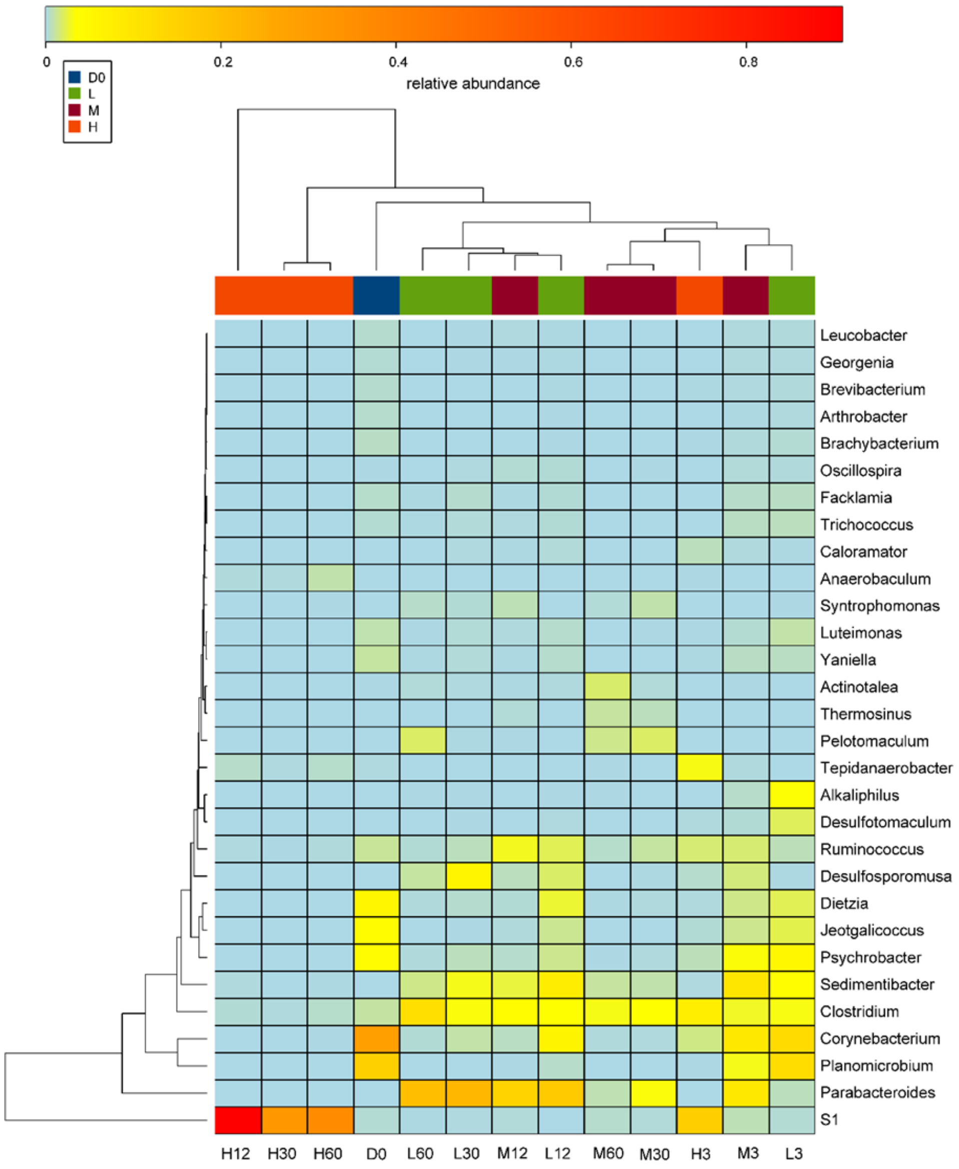

Figure S4 Heatmap showing the relative abundance of 30 most abundant genera during anaerobic digestion at 20°C (L), 35°C (M), and 55°C (H). D0 represents the initial material.

References:

1. Aminov, R. I.et al. Development, Validation, and Application of PCR Primers for Detection of Tetracycline Efflux Genes of Gram-Negative Bacteria. *Applied and Environmental Microbiology*. **68**, 1786–1793 (2002).

2. Aminov, R. N., Garrigues-Jeanjean N. & Mackie R. I. Molecular Ecology of Tetracycline Resistance: Development and Validation of Primers for Detection of Tetracycline Resistance Genes Encoding Ribosomal Protection Proteins. *Appl. Environ. Microbiol.* **67**, 22–32 (2001).

3. Ng, L. K.; Martina, I.; Alfab, M.; Mulveya M. Multiplex PCR for the detection of tetracycline resistant genes. *Mol. Cell. Probes* **15**, 209–215 (2001).

4. Frank, T., Gautier, V., Talarmin, A., Bercion, R. & Arlet, G. Characterization of sulphonamide resistance genes and class 1 integron gene cassettes in Enterobacteriaceae, Central African Republic (CAR). *J Antimicrob Chemoth*. **59**, 742–745 (2007).

5. Xu, J. et al. Occurrence of antibiotics and antibiotic resistance genes in a sewage treatment plant and its effluent-receiving river. *Chemosphere* **119**, 1379–1385 (2015).

6. He, L. Y. et al. Dissemination of Antibiotic Resistance Genes in Representative Broiler Feedlots Environments: Identification of Indicator ARGs and Correlations with Environmental Variables. *Environ. Sci. Technol.* **48**, 13120–13129 (2014).

7. Steinberg, L. M. & Regan, J. M. Phylogenetic comparison of the methanogenic communities from an acidic, oligotrophic fen and an anaerobic digester treating municipal wastewater sludge. *Appl. Environ. Microbiol.* **74**, 6663–6671 (2008).
